# Lessons learned from implementing alternative rites in the fight against female genital mutilation/cutting

**DOI:** 10.11604/pamj.2019.32.59.17624

**Published:** 2019-02-04

**Authors:** Ernst Patrick Graamans, Tara Rava Zolnikov, Eefje Smet, Peter Ngatia Nguura, Lepantas Charles Leshore, Steven ten Have

**Affiliations:** 1 School of Business and Economics, Change Management, Vrije Universiteit, Amsterdam, Netherlands; 2 School of Health and Human Services, National University, San Diego, California, United States of America; 3 Programme Management, Amref Health Africa, Leiden, Netherlands; 4 Anti-FGM/C Center of Excellence, Amref Health Africa, Nairobi, Kenya; 5 Alternative Rite of Passage/Water, Sanitation and Hygiene, Amref Health Africa, Nairobi, Kenya

**Keywords:** Female genital cutting, non-adherence, rites of passage

## Abstract

**Introduction:**

historically, programs aimed at making communities abolish female genital mutilation or cutting (FGM/C) consisted mainly of awareness campaigns on sexual reproductive health and rights and the enforcement of newly implemented laws. These types of programs or interventions appear to be only partially effective and sometimes yield unintended results, such as actually strengthening commitment to FGM/C or transforming it into a secret practice. A newer approach to change that is intended to account for the cultural meanings ascribed to FGM/C are alternative rites of passage (ARP). Amref Health Africa started adopting this approach in 2007. Since then, by a trial-and-error process lessons have been learned, that will be reflected upon in this paper.

**Methods:**

desk research was conducted on organizational data regarding all Amref Health Africa's efforts to end FGM/C. Ninety-four in-depth formal interviews were held with members from Maasai and Samburu communities in Kenya targeted through maximum variation sampling. And participant observation of significative events as well as daily pastimes took place during school holiday season at the end of 2016. Furthermore extensive informal talks were held with project donors, activists, journalists, members of other non-governmental organizations, members of community services organizations, local government officials, high-ranking Dutch and US diplomats and senior members of the Anti-Female Genital Mutilation Board, which is part of the Kenyan Ministry of Public Service, Youth and Gender Affairs. On the basis of these data a framework on different positions on FGM/C was developed and published in early 2018. By reviewing the data again from a particular change management and public health perspective, by peer-debriefing within a multi-disciplinary research team and by explicating the lessons learned this paper adds to an overview that is of crucial importance to practitioners working to end FGM/C.

**Results:**

risk of exclusion, perceived loss of cultural identity, changing meanings ascribed to cultural practices, lack of precise knowledge about subjective (sexual) experience and negative stereotyping are reasons not to adhere to anti-FGM/C programs. Areas of concern are the role confusion with following-up on policing, perceived outsider interference and the intended prolonging of the transition phase into womanhood not being explicated and embedded with ARP. Aspects to enhance to lever change more effectively are education and school curriculum development, male involvement, new stylization of love relationships, monitoring and evaluation and inclusive aspects of religion.

**Conclusion:**

changing a culturally embedded practice such as FGM/C is inherently complex. Because the cultural meanings ascribed to this practice are also evolving, any intervention that is effective at present might become superfluous in the future. A holy grail approach to change simply does not exist. Change needs to be levered in a variety of ways while working on the alignment of all these efforts by regular and thorough quantitative and qualitative assessments of effects and side-effects and reflections on lessons learned.

## Introduction

Female genital mutilation or cutting (FGM/C) is recognized worldwide as a harmful practice and is suggested to contribute to an array of adverse health effects, such as traumatic bleeding, infections, urinary problems, psychological trauma and even death [1-3]. However, reliable data on prevalence and the precise causal effects, related to type of procedure of cutting for example [1, 4] or how best to effect change [5], have been and continue to be difficult to determine. It is estimated that approximately 200 million girls have undergone FGM/C [3]. While FGM/C is an illegal practice and the prevalence is steadily falling [2], Kenya is one country that continues to suffer from high rates of FGM/C amongst some of its communities. Among the Maasai and the Samburu, the proportion of women who have undergone this practice is reported to be around 78% and 86% respectively [6]. Several non-governmental organizations (NGOs) are implementing alternative rites of passage (ARPs) to address these prevalence rates in these communities. However, at the same time, voices are challenging these organizations for the use of ARPs and the lack of evidence regarding effectiveness [7, 8].

**Alternative rites of passage:** historically, interventions against FGM/C were aimed at making people aware of the negative health effects, inculcating them with 'western', modern and/or Christian values and changing legislation [9]. Being aware of setbacks of the past and to avoid another 'culture clash' required that a different type of intervention to be developed, which was called the alternative rite of passage (ARP). However, prudence is called for going forward, because ARP, its design, implementation and impact or effects, are still underresearched [10]. Amref Health Africa, an African-based health organization, employed this intervention in 2007 in nomadic communities in Kajiado County, Kenya and then eventually expanded the program to other countries (Tanzania) and other communities (Samburu) where FGM/C is known to be a common practice. Firstly however, the meaning of what ARP exactly entails also needs to be clarified. For the purpose of intelligibility and marketing, the explanation given to donors and the general public was that ARP is a straightforward alternative to 'the cut', whilst everything else stays the same. Girls who participated in ARPs were said to be saved from having to undergo FGM/C. However, this oversimplification of what ARP is intended to achieve and the way it is supposed to be achieved makes embedding ARPs within existing social arrangements even more difficult; in fact, the meanings ascribed to ARPs by community members themselves and girls that participate in them do not seem to justify the assumption that they are saved from FGM/C after having gone through an ARP. The sweeping generalization of what ARP entails obscures the fact that these programs were never intended to replace 'the cut' for an alternative ritual, which essentially marks the transition to womanhood and thereby makes girls eligible for marriage.

On the contrary, the ARP designed by Amref Health Africa was also supposed to be used as a platform to bring an end to child marriage while reducing school dropout and encouraging educational attainment. So actually the adage should be 'stop the cut and some of its outdated cultural implications too'. This ambition that involves multiple goals has, willingly or not, created the situation where a health organization with a good reputation and restricted scope related to health issues became involved in a multifaceted change initiative that some would label a culture change intervention, or even a 'social engineering experiment' [7] of which the consequences are not fully understood yet. Both of these characterizations are problematic, first of all for Amref Health Africa itself. Amref Health Africa does not intend to change an entire culture, only a few specified practices which might be labeled 'cultural' [11]. Note that culture itself is a problematic notion in explaining behavior or the tenacity of certain practices [7, 12]. As said, one researcher uses the term 'social engineering experiment' in relation to ARPs [7], which highlights the fact that some things are still unknown or unclear and further research is warranted. Although this characterization is made in relation to a study on ARP as implemented by another NGO, the term 'social engineering' can have a slightly negative connotation, mainly because this wording can subtly imply this change effort is conceptualized as initiated top-down or outside-in and is implemented rather mechanistically [13]. However, Amref Health Africa was trying to avoid both of these things in the design and implementation of its ARPs. The whole point of the program was to empower affected subgroups within practicing communities that wanted to resist FGM/C but did not have a feasible way of doing so. That said, ARP can somewhat be conceptualized as an experiment since it indeed came about by a trial-and-error process without a clear theory or framework of culture by which to guide it.

As earlier research indicates, although for many the practice of FGM/C signifies a transition from girlhood to womanhood, it simultaneously functions as more than just a rite of passage [14-17], though this particular function is the focal aspect by which ARPs are intended to lever change. To complicate things further, several Maasai and Samburu women had expressed that the practice of FGM/C does not feel like a rite of passage at all [14]. These women have pointed out that they still must greet elders by bowing their heads after having undergone FGM/C, to receive blessings, which is also perceived as a symbolic gesture signifying unequal power relations that perpetuate the patriarchy. For comparison, young boys are exempt from this convention after circumcision. So, in what way, do women get the full and equal respect as an adult after this rite? To these women FGM/C explained as a rite of passage feels like a sham and conceals the real reason, which simply is control and oppression by men over women. To bring some oversight into these layered and complex dynamics some of the authors of this paper created a framework that reviewed the different ways Maasai communities around Loitokitok and Magadi, Kajiado County and Samburu communities around Wamba, Samburu County accounted for FGM/C and examined how these actions related to the underlying behaviors and practices [14]. Drawing upon the same data set on the basis of which that framework was developed and consequently published in *Culture, Health & Sexuality* in early 2018, this paper more specifically addresses the implications for the practice of designing and implementing interventions aimed at ending FGM/C, such as ARP which is the main focus. In order to do so the data have now also been looked at through a public health and change management lens.

## Methods

**Ethics statement:** the research protocol was approved by the Ethics and Scientific Review Committee (ESRC) of Amref Health Africa in Kenya (REF: AMREF-ESRC P277/2016) on October 27, 2016. This Committee is accredited by the Kenyan National Council for Science and Technology to perform the ethical review for research protocols involving human subjects.

**Desk research:** we gained access to all organizational data relevant to Amref Health Africa's efforts, past and present, to combat FGM/C. These include unpublished reports of a baseline survey and end term evaluation on the effects of Amref Health Africa's ARP, and some confidential correspondence between important stakeholders of anti-FGM/C programs. We also studied a draft version of a new Maasai Constitution that is supposed to be more aligned with Kenyan law on matters of sexual and reproductive health and rights (SRHRs) for example.

**Interviews:** over a one-month timeframe at the end of 2016 the principal investigator conducted 94 formal in-depth interviews with community members that were targeted through maximum variation sampling. This sampling process continued until saturation was achieved. The sample consisted of children, adolescents, adults and elderly of both genders and fulfilling a variety of community roles, both traditional and contemporary. In preparation of and throughout this research project the principal investigator also had numerous informal talks with other stakeholders, such as project donors, activists, journalists, members of other NGOs, members of community services organizations (CSOs), local government officials, high-ranking Dutch and US diplomats and senior members of the Anti-Female Genital Mutilation Board, which is part of the Kenyan Ministry of Public Service, Youth and Gender Affairs.

**Participant observation:** in order to triangulate the data collected through desk research and formal interviewing and to gain a better 'feel' for what is at stake for people in communities still practicing FGM/C the principle investigator conducted participant observation at several sites. To give an impression Table 1 provides a rough overview of these observations that were worked out in an expanded observation protocol.

**Table 1:** participant observation

Socio-cultural situation	Place	Actors	Activities
Three-day stay at traditional manyatta	Manyatta and surrounding area	Morans from ± 25-35 years old; children; mothers	Singing and dancing; ‘making stories’; helping to repair the kraal; witnessing the slaughter of a goat; preparing *supu*; minding cattle; visiting nearby manyattas
Two-day stay at homestead (location A)	Traditional homestead	Mother; children; oldest son accompanied the researcher	Weeding to protect crops; inspecting arable land; making *ugali*; milking cows; informal talks with agricultural labourers
Two-day stay at homestead (location B)	Traditional homestead	Father; mother; children; father accompanied the researcher	Long bush walks; preparing *supu*; ‘making stories’; drinking sodas in town with other morans; inspecting arable land
Preparatory meetings for upcoming ARP	Homestead with brick walls and corrugated metal roofing	Woman involved in organizing the ARP; male primary school teachers; Amref ground staff	Praying; discussing logistics of getting the girls from their villages to school premises, security issues and deployment of tasks
Three-day ARP program + preparations (location A)	School premises; agricultural area	Girls and a few boys; organizers; educators; facilitators; government officials; journalists, camera crews; Amref staff; anti-FGM Board members; former circumcisers; morans; cultural elders	Full ARP program consisting of classroom educational sessions, procession through town, beauty pageant, theatre plays, ‘candle night out’, final ceremony, stakeholders’ speeches, etc.
Three-day ARP program + aftermath (location B)	School premises; remote area; no agriculture / pastoralist	Girls and a few boys; foreign delegations; facilitators; government officials; journalists; security officers; Amref staff; anti-FGM Board members; former circumcisers; morans; cultural elders	Idem
Two-day ARP program (location C)	Traditional manyatta close to urban area	Girls and a few boys; foreign delegations; government officials; journalists; security officers; Amref staff; anti-FGM Board members; former circumcisers; morans; cultural elders	Idem + video show ‘FGM in reality’; blessings by cultural elders together with foreign delegates!
Full day on local market	Daily market at urban area	The aim was to talk to young men either informally or via in-depth interviews	Assisting in a small permanent shop selling vegetables, eggs, flour, sugar and batteries; informal talk
Half day on cattle market	Small-scale cattle market	Morans; elderly men; shepherds	Informal talks under a tree
Midnight open-air Pentecostal service	Manyatta	Pastor / preacher; men and woman; young and old; mostly shepherds from this manyatta or manyattas close by	Praying; singing; dancing; holding hands; listening to the preacher; waiting two hours in the dark for the generator to start
Two-day boy circumcision (*emorata*) ceremony	Traditional homestead	All members of the community and visitors from nearby areas	Congratulating the young boy before and after circumcision; drinking traditionally brewed honey beer; ‘making stories’; traditional singing and dancing; disco-dancing

**Data-analysis:** the data were assembled and integrated using qualitative methods based on both a discourse-analytic approach to talk and text [18-20] and a cultural psychological approach to embodied practice [12] in order to, amongst other things, create a framework that helps to better understand the very different and at times opposing, positions people can adopt on FGM/C. As said, this framework was presented in early 2018 in *Culture, Health & Sexuality* [14]. In that paper a general call is made to adopt more contextualized and holistic approaches to change if the aim is the total abolition of all types of FGM/C, but without going into much further detail. Looking at the same data set from a public health perspective as well as a change management perspective and by conducting extensive peer-debriefing sessions in the period thereafter amongst the authors of this paper, we came to an overview of the lessons learned from implementing ARP in the fight against FGM/C. However, we stress that these lessons are not only relevant to the design and implementation of ARPs. ARP, we contend, is just one possible lever in a complex dynamic of change. So what follows are the reasons for non-adherence to anti-FGM programs, the areas of concern and the aspects to enhance, that we have identified based on our data and further substantiated by literature.

## Results

**Reasons for non-adherence to anti-FGM/C programs:** first, girls that refuse to become circumcised in many areas run a significant risk of becoming expelled from their respective communities. They will have to endure bullying, often from their already circumcised peers [21]. This can become such an unbearable torment that from the girl's point of view, surrendering and getting circumcised really is the only option. It would be a mistake, however, to consider this a 'voluntary' choice to get circumcised. It is not a choice, since there was no realistic alternative option. This situation means that in the design of any intervention aimed at durably ending FGM/C, a variety of support groups, safe houses, or community policing structures must be firmly put in place right from the start. Women that stand against cultural practices take a risk and need to know where they can find support or protection in case they encounter repercussions. Second, if people feel their culture is under attack, resistance is a near certainty. This does not mean that people are averse to change per se but change perceived as being initiated from individuals who are not part of their social arrangement almost automatically ignites some type of resistance or sabotage [14]. For example, religious missionaries have experienced many devastating setbacks while trying to make people abandon indigenous practices, such as FGM/C [22]. This means that both in the design and implementation of any change of a historically-imbedded cultural practice, involvement of the targeted community should be central. Any attempt to change even the smallest aspect of an indigenous arrangement will fail if community members themselves are not involved from the start. These community members, previously held in a cultural arrest, can now become “change champions”, empowered and emboldened to create change in their community. This immersion can be done through a variety of ways, such as education, financial incentives, access to resources, etc.

Third, consensus of what FGM/C signifies does not exist. Practicing people account for it in a variety of ways, even within a single community [14-16]. To complicate things further, there is no standard set of practices -in fact, there are four types of cutting in FGM/C- and also the meanings ascribed to the practice have changed over time [17]. This situation suggests that an implemented intervention that works one day will not necessarily work in the future. This fluidity means that each intervention needs to be continuously assessed and re-evaluated as to whether progress is being made to eliminate FGM/C. Fourth and as mentioned above, FGM/C is classified into four types of procedures [3]. The amount and type of tissue cut varies, as do related health risks. Although FGM/C is often regarded as a practice that dampens sexual desire in women, it is unclear how it exactly affects the subjective experience of sex [5, 23]. Though, outcomes related to health and sexual desire likely depend on the type of cut, automatically assuming decreased sexual pleasure in all women or exaggerating negative effects of FGM/C can undermine the credibility of the messenger, the intervention and the rationale for change. This situation can create doubt, wherein women who have undergone the procedure could say otherwise and have women disbelief the information regarding anti-FGM/C. Secondly, the argument might make women who have undergone FGM/C oppose and feel stigmatized. This information suggests that the interventions need to be based on precise and contextualized knowledge and take into account the position and feelings of different actors. And finally, the premise of any intervention aimed at ending FGM/C should be that all people, also those that practice FGM/C, love their children [24]. Facilitating open conversations about how loving relationships of all kinds (e.g. friendly, parental, conjugal, etc.) are shaped and can be re-shaped from within the community might be one of the most fundamental levers of change. Insinuating, however subtle, that people who practice FGM/C are lacking in love and empathy, blocks this opportunity for change.

**Lessons learned: areas of concern:** ARP, in general, should be considered a fluid process, in which changes are consistently being made, evaluated, and presented. As mentioned before, ARP could be a catalyst for change but the outcomes within a community are difficult to predict and may vary for various reason (e.g. context, time, the way it is implemented, etc.). Thus, current lessons from the field have encountered some areas of concern as well as opportunities for change. Areas of concern that affected ARP response (e.g. Maasai and Samburu in Kenya) included understanding the role of policing, addressing suspicion about perceived outsider influence and indistinctness related to what the alternative rite entails.

**Policing of Female Genital Mutilation:** in 2011, the Kenyan government passed the Prohibition of Female Genital Mutilation Act [25], in which FGM/C is constituted as a serious crime. This crime, punishable by the law, includes not only performing the actual cut, but also possession of tools for cutting, the use of derogatory language in relation to uncircumcised girls and failure to report commission. That said, enforcement of the law can be complicated, especially in remote areas, so community policing structures, the Nyumba Kumi initiative, were created by the government to help enforce these and other rules [26]. However, some confusion and misunderstandings exist among the community about institutional roles as exemplified in a quote by a Maasai man, “*If I hear that a girl is about to be circumcised, I will call Amref. They will come and put them (the perpetrators) in jail*.” (P1.2). Obviously, it is not Amref Health Africa that puts people in jail. Amref Health Africa has invested a lot of time and energy to build rapport and gain excess into communities. In general, the organization has a good reputation and their work on health issues is widely appreciated, except that despite these beneficial relationships with communities, there is still some hesitation from people who continue to favor FGM/C. Thus, Amref Health Africa, by some community members wrongfully perceived as being an enforcer of the law, in some situations has to wager the fragile link to community members, which is crucial for their anti-FGM/C work. Amref employees are aware of this tension: *"There are risky cases where we intervene and call in the police, but our main work is talking to the communities. But of course, when girls are married off or anything we intervene, but not directly. We want to hold the departments accountable, the children's department of the government for example. But it is very tedious. Sometimes they don't have vehicles, so we say: “Come on we'll take you there with our vehicle”. It is frustrating if they don't have the resources. We don't like taking that responsibility. But sometimes we are helpless. We don't believe in arrests, we believe in talking and negotiating. But if the life of a girl is at risk our destiny is jeopardized, then we don't mind helping and crossing the boundary if that way the girl is saved. Once in a while, we unloose our reputation for a day or two. But our key way is known: it is talking and initiating dialogue. But if need be, in very desperate cases, if law enforcement needs support, we help them. But we prefer to give them fuel (so they can drive with their own vehicles)."* (A1.19). This situation is precarious in that the individuals who are trying to create change are also viewed as complicit in putting their community members in jail. Although it is beholden on Amref Health Africa to report cases of crime and it is perfect socially and ethically responsible conduct on the part of any NGO involved to act in the manner as described above, it has an unintended consequence. Again, this situation can contribute to increased cases of FGM/C instead of decreasing the practice because people will be actively avoiding the information promoting ARP, or Amref Health Africa in general. Also, it increases the risk that practices will be done in secrecy which makes it even more difficult to control. Moreover, it will influence the trust relationship between the community and Amref Health Africa's field workers and researchers to signal imminent cases or to get reliable data about the prevalence -or changed rates-of female circumcision.

**Suspicion and perceived outsider interference:** since Amref Health Africa was aware from the beginning that change perceived as initiated by outsiders will not be successful or, as mentioned above, is a reason for non-adherence to any anti-FGM/C program, working on community buy-in played a large role in delineating their ARPs. However, although the intent was there, in practice accomplishing this buy-in appeared to be challenging, especially in those community members that were already suspicious. FGM/C is a way of incorporating someone into the fold as a matter of identity and belonging, as this statement of a Maasai cultural elder signifies:*"Wherever you go, go visit the Maasai in Kaijado, Laikipia or Samburu, irrespective of where you come from, the Maasai culture states that all the girls, all the women and all men are to be circumcised. No exceptions are made. So, it is very hard for me to leave that culture. And I see no reason to change. Go and tell the Luo's that from today onward they should circumcise their girls, something they have never done before, they will not do it. Similarly, if you tell Maasai not to circumcise their girls, something we have done since a very long time, it is very hard,"* (P13.4). For example, elders were suspicious about who was behind the ARP program and why outsiders were trying to change their culture. Community members perceived these influences as changes to their culture and in turn, questioned the program. *"This idea that circumcision is bad, where is it coming from? It is not an idea from us. It is an idea from outsiders. It is not coming from the Maasai, but from somebody else. You are now trying to take me away from my culture to somebody else's culture. You see, there are many communities within our country that do not practice circumcision of the girls. We are not like these communities. And now you are trying to make us like them,"* (P7.3). Although there is no doubt that Maasai identity is continuously challenged by outside influences, change is also largely stimulated through their own members. Using these Maasai as champions of change is an important asset in Amref Health Africa's ARP that was designed to curb suspicion by implementing a community-led program. As a way of uplifting the ceremony, which is not yet embraced by the whole community, VIP guests can serve as a wider token of support, encouragement and endorsement for the girls and other inside people involved. At the same time, however, the whole idea of ARP as a community-led and indigenous initiative can be jeopardized by the observed spectacle of non-Maasai or non-Samburu VIP's, foreign journalists, camera crews and researchers flocking to the final ARP graduation ceremony. It evokes an undeniable picture of foreign intervention or civilization offensive, whereby some people try to manipulate attitudes and behaviors of others through ideological means [27]. At several ARP ceremonies, it was observed that all performances were centered around the foreign guests. Those same guests later had lunch in separate quarters, where the food served was of better quality and more varied. It goes without saying that those VIPs were important stakeholders and well-wishers of the project and the ARP ceremony is also about getting publicity for the project. However, it does detract from an image of the ARP as community-led for those who are already suspicious of the origin of the idea.

**Separation, transition and incorporation:** ethnographer and folklorist Van Gennep made the case that rites of passage can be subdivided into and consist of rites of separation (e.g. child to parent dyads), rites of transition (e.g. childhood to adolescence or emerging adulthood), and rites of incorporation (e.g. marriage) [28]. Hence, rites of passage may be stretched over a prolonged period of time. Theoretically, in the design of alternative rites of passage, these three aspects or phases should also be accounted for and parallel the original rite. From observations during research in the field, it appeared that these aspects have not been thought out or explicated. It has been suggested that ARPs might prolong the transition phase from girlhood to womanhood. Girls that participated in ARPs indicated that they do not feel like women, neither are they considered as such by the community. It is also unclear what type of pubescent or adolescent behavior is acceptable or expected of them, apart from being labeled and acting like 'school girls.' Additionally, returning home to an unchanged social arrangement, could trigger similar behavior in both parents and girls that make it seem like the rite did not have any affect (e.g. changed her status in the community). One suggestion could be to put more effort into including parents and caregivers in the ARP to further add a parental support dimension to the program. Thus, ARPs need further refinement because of their lack of comprehensiveness. Looking at the 'original' rite, separation, transition and incorporation (e.g. marriage and motherhood) are three significant stages that are currently not focal points of ARP programs. Simultaneously, while rejecting FGM/C, ARPs also reject the practice of child marriage, which is of course a crucial difference with what is implicated in the 'original' rite. The crossover between one rite to the next is not consistent and these inconsistencies also lead to the difficulties in program adoption. That said, it is not yet clear which elements should and could be levered to account for all three aspects of a rite of passage to be able to more thoroughly embed ARP within a contemporary social arrangement that is acceptable to Maasai and Samburu. Further research is warranted in that regard.

**Lessons learned: aspects to enhance:** lessons learned from the field provided insight into aspects of ARP programs that should always be included and/or enhanced in the design and implementation of the intervention. These areas include education on circumcision and SRHRs, male involvement, religion as supportive element, awareness on relationship development and monitoring and evaluation. Together, these areas are likely to enhance outcomes of ARP and promote program success.

**Education:** education is a main component of ARP; in fact, education appears to be seen as the solution to almost everything throughout the ARP program. Even more so, it is apparent that lack of knowledge is thought to be the main problem causing the perpetuation of FGM/C. *"I do not blame my parents, because they never had the knowledge. Nobody could tell them. The church could not speak about it. Also, the government did not do at that time. So they had to follow the culture as they were taught."*(P19.6). A slogan often heard while attending ARPs is 'Education is circumcision of the brain', meaning that education can transform girls into women and not circumcision. Education is construed as a form of female empowerment that is likely able to equalize the male-female balance. The assumption behind increased education is that women who are educated are aware of their rights and will be able to better claim and defend these rights for themselves and their daughters/children. Second, it may reduce their dependency and give them more economic stability, as women have their own income. One participant confirmed the role of education promoting women's empowerment:*"You know, in my community the voice comes from the elderly, the elderly men who are the age of my father now. They are the ones who control everything in the community. The young warriors and the upcoming men, they just have to follow their culture. It is only if one is educated that we (as women) can stand and say: 'No, our fathers did it like this, but now there is another direction'."* (P19.6)*.* Because morans, or young men of the traditional shepherd-warrior class, often do not attend school and instead focus on occupations to sustain their future families (e.g. animal husbandry), the aspect of education can fall short with this important population. Education has brought about major lifestyle changes and areas of concern that may not be overlooked as well. As said, school fees need to be paid for, often by selling cattle, children leave for boarding school, but more importantly children come home with the desire to become a lawyer, doctor or pilot. None of the children interviewed said they wanted to become a moran or shepherd and raise their kids in a traditional manner. The pastoralist lifestyle is becoming, at best, something to fall back upon if everything else fails and herding is becoming at best an enjoyable pastime, and not something to be considered as a full-time profession. However, it is possible that inflated promises about what education brings may lead to disappointment. And this sequential disappointment can lead to a setback in progress being made in the work against FGM/C or the adoption of ARP. Not everybody will or can become a lawyer, pilot or doctor. School fees, especially for higher education, are still a big problem for many encountered in our field work. That said, education remains an important tool against FGM/C.

**Importance of male involvement:** at the same time, ARPs should be more inclusive to boys and men. *Morans*, or boys who undergo rites of passage to become men, and traditional values of warriorhood in the Maasai tribe are admired and feared at the same time, but also seen as something of the past. From an integral change approach, it is important not to overlook their concerns and stakes. Doing so can lead to setbacks in the future. They might feel ignored and want to maintain older practices, such as working to re-introduce FGM/C. One Maasai school boy addressed the difference between traditional morans and school boys his age in the following manner: *"You see all these boys here dressed traditionally (for the ARP ceremony)? They are just school boys. A real moran doesn't come to these gatherings. They don't associate with the people here. They go by themselves. A real moran does not dress like these boys. They wear only one shuka; just on one side, with a knife here and a spear. They (the boys here) are just acting like morans. It is just for the ceremony that they dress traditionally. For the show. Actually, you can only be called a moran when you are a real Moran. When you live in bushes. When a lion roars, they are there to make sure that it is killed."* (P13.1)*.*

**The role of religion:** the role of religion in ARPs was recently examined more closely and it was found that ARPs run by a Christian NGO were couched in terms such as salvation, transformation, morality and Christian symbolism [7]; that said, this particular study did not focus on the role of religion in ARPs facilitated by secular NGOs, such as Amref Health Africa. However, our study confirmed the presence of Christian terminology and practices, such as prayer in ARPs, although maybe not to the same degree and in a different (more modest) manner [14]. Because religion is deeply integrated with Kenyan daily life, it appeared to be a significant reason why people were accepting ARPs and was suggested to be used as a 'tool' against FGM/C. A young Maasai girl used it in an attempt to convince her father against undergoing FGM/C: *"I read verses from the Bible…in the Bible, I have never heard about girls who have been circumcised. It is just an idea of men. So, I told my father that I don't like it,"* (P13.3). It is important to note that using religion as a tool is a very sensitive and even hazardous inroad to try and effect change. Hesitation to include religion stems from a concern that overly Christianized ARPs could be used as tools for proselytization. Also, not all Maasai designate themselves as Christian, or Muslim for that matter. Maasai also have their own indigenous religion that is still practiced in many regions. Christianity as such is not indigenous to Maasai culture and might even be off putting to some who consider Christianity and Islam as man-made religions to be distrusted as foreign to their culture. That said, for non-faith-based or neutral NGOs, such as Amref Health Africa, religion does not necessarily have to be bypassed or ignored entirely. It depends on FGM/C perceptions in a region and how it relates to religion. If the religious discourse is very strong, it better not be ignored. In fact, it is probably better to include religion in an integral approach to change. For example, many participants drew inspiration from the Bible in a way that enabled them to cope and deal with adversity. Religion is an undeniable factor and lever in relation to FGM/C; however, its role must be continuously and critically evaluated within each particular context and region.

**Stylization of love as a lever of change:** after a careful review of the research conducted in the field and evaluating lessons learned, perhaps the least recognized lever of change focuses on love; love is most apparent in that parents who love their children want the best for them and do not want them to suffer. As the meaning of FGM/C is changing, this painful procedure will increasingly go against paternal instincts regarding protection of their children [29]. Moreover, Maasai and Samburu women and men often find themselves taking part in this practice against their own will. One (former) circumciser confided that with her own children, she chose to cut less tissue: *"(Between) my daughters and other girls, there was a difference. With my own daughters, I was not cutting everything, only a small part. So, I made it less painful for them. But this is not possible for the other girls. The mothers take me and bring me to their bhomas (houses). They are there and give me instructions on how to do it, how much I should cut. They tell me do it like this, remove this, take this like that. So, there is a difference. My daughters were getting healed quickly. They were not bleeding a lot,"*(P7.3). In fact, she later confided that when she cut the girls and heard them scream she had to suppress her own tears and concentrate on holding her trembling fingers still. This scenario was recalled in more accounts by former circumcisers. Another story by a cultural elder described how he had ordered an immediate end to the procedure because he could not bear to hear his daughter scream and suffer. All these accounts show that underneath the surface Maasai and Samburu themselves are not comfortable anymore with a tradition that seems to be holding them back in many ways.

**Continuous monitoring and evaluation:** ultimately, any intervention with the aim of encouraging people to abandon a historically-embedded cultural practice will likely suffer from some types of side-effects or negative unintended consequences. In the search for effect and realizing that one-sided approaches do not work, Amref Health Africa has moved beyond its familiar medical paradigm to cover the encompassing aspects of FGM/C. The NGO has realized that by doing so, they run the risk of becoming entangled in issues that are beyond their original vision and mission statement. By pulling on more change levers comes an increased responsibility. Unforeseen consequences, if negative to the well-being of community members, first and foremost for the girls themselves, need to be acted upon. To be able to do that strong monitoring and evaluation (M&E) structures need to be put in place. Again, this confronts NGOs with all kinds of practical (who is doing the monitoring and by what means?) and ethical (how to guarantee privacy?) challenges. However, fighting against FGM/C is not for the fainthearted and solutions must be sought after. Presently, initiatives are undertaken by this NGO to implement more thorough M&E structures to be able to follow-up on girls after they have participated in an ARP.

## Discussion

Based on literature and further substantiated by our own findings we have identified five salient reasons for non-adherence to anti-FGM/C programs. First, as other researchers have also noted [15, 16, 21], there is the problem of the oftentimes real risk of exclusion when adhering to such programs. Second, a perceived 'culture clash' when programs are seen as a form of outsider interference, might actually strengthen the commitment to practices such as FGM/C instead of weaken it [22]. Third, because meanings ascribed to FGM/C are continuously changing [17] and are multi-faceted [15, 16] programs can lever change effectively at one point in time but be superfluous or even contra-indicated at another point in time. Programs also need to be contextualized to a specified target community, since there simply is no one-size-fits-all or holy grail approach. Fourth, lack of knowledge about the precise effects of FGM/C or exaggeration of these effects in order to create a sense of urgency, might actually lead to the messenger losing credibility. As yet for example, we simply do not know enough about the effects of FGM/C on sexual experience [4, 23]. And last, negative stereotyping of people in FGM/C practicing communities by those trying to affect change will block any possibility of change [24].

We also have identified three main areas of concern that NGOs working in the field need to be aware of and deal with. First, the order not implying sequential importance, they will have to strike a difficult balance between dialogue and policing. Second, related to our second reason for non-adherence, any attempt to change by ARP or otherwise that is perceived as initiated by outsiders will likely lead to suspicion in those that already feel that their culture and identity are under threat. The, at times overwhelming, presence of international delegations, whether consisting of high-ranking government officials, journalists accompanied by camera crews, donors or researchers like ourselves, at the final ARP graduation ceremonies subtly and not so subtly jeopardized the image of ARPs as being community-led. Also, bridging to our third area of concern (what exactly is a rite of passage), the ARP as an event open to everyone that is interested does not mirror the intimacy and feel of a rite of passage, as some community members themselves have noted in follow-up sessions after our initial research. And ARP as it is implemented now implies the prolonging of the transition phase to womanhood, which creates new challenges to young girls that need to be acted upon. Lastly, we have identified several aspects to enhance. First, education is and will remain an important aspect in the fight against FGM/C and school curricula can be further developed to emphasis SRHRs and matters of Maasai identity and culture. Second, the involvement of boys and men needs to be strengthened considerably. FGM/C is not just a women's issue, but directly effects men as well. Third, the role of religion should be addressed. Religion can be constructively used as a lever of change. However, ARPs should not be used as a tool for proselytization. Fourth, FGM/C has everything to do with the way love relationships are shaped, both conjugal as well as parental. Explicating love problems as well finding new ways of shaping sexuality, intimacy and care provide interesting and underused levers of change. Lastly, further strengthening M&E structures will help to be able to follow-up on girls who participated in ARPs. Efforts have been undertaken to strengthen M&E, but improvement is warranted.

What could be considered a weak point of this study by those that seek 'hard' quantifiable data, is in fact an inevitable aspect of a study on dynamics of change. We contend that although our findings are not substantiated by numbers, they do provide some useful insights and tools to further improve initiatives with the aim of bringing an end to FGM/C. However, change practitioners should be careful with generalizing our findings too easily to other communities that perform this practice for a different set of reasons, for example more as a guarantee for virginity (as can be the case with infibulation or pharaonic circumcision). ARP is not and was never intended as a one-size-fits-all kind of intervention. All interventions should be contextualized to the target community and be based on an a priori assessment of the positions different stakeholders within and around the community take up.

## Conclusion

Changing a culturally embedded practice, such as FGM/C, is complex. Advocates seeking to abolish the practice are mobilizing people to collaborate in programs to stop 'the cut', such as ARP. However, because of the complexity that comes with changing FGM/C, difficulty in measuring change and the continual need to revise and adapt to challenges encountered during program implementation, project success is yet not visible. At this point, the benefit of working and assessing direct changes related to FGM/C has provided some insight and direction regarding limitations and focal points that will possibly work and resonate with the target audiences of the interventions to ensure more successful program result and decreased rates of FGM/C.

### What is known about this topic

- Over the years governments and NGOs have put in considerable effort into making people in FGM/C practicing communities abandon this practice on the basis that it is a violation of human rights and harmful to women's health;
- The effects of these programs, interventions or campaigns are difficult to assess, but it appears that success so far has been limited, especially in light of the expressed commitment to a vision to end FGM/C entirely by 2030;
- More recently NGOs are implementing ARPs in the fight against FGM/C in an effort to lever change in a way that accounts for meanings ascribed to this practice by the communities itself. However, the effects, side-effects and the way ARPs are designed and implemented are as yet underresearched topics.

### What this study adds

- Based on empirical data collected around ARPs conducted by Amref Health Africa and intensive peer debriefing within a multi-disciplinary research team this study positions ARP, not as a holy grail intervention, but as a possible lever within a wider dynamic of change. This study integrates cultural psychological, medical anthropological, public health and change management perspectives in order to contribute to the design and implementation of more effective interventions;
- The lessons learned from implementing ARPs are relevant to all that work in the field of SRHRs, since it has become evidently clear that one-sided approaches do not work;
- The findings underscore the importance of paying attention to and accounting for the implicit meanings that a culturally embedded practice such as FGM/C can have and the importance of continuously evaluating how these meanings are evolving over time in order to re-align change interventions to the reality in the field and for the people concerned.

## Competing interests

The authors declare no competing interests.
